# Low-intensity ultrasound combined with allogenic adipose-derived mesenchymal stem cells (AdMSCs) in radiation-induced skin injury treatment

**DOI:** 10.1038/s41598-020-77019-9

**Published:** 2020-11-17

**Authors:** Zeinab Hormozi Moghaddam, Manijhe Mokhtari-Dizaji, Mohammad Ali Nilforoshzadeh, Mohsen Bakhshandeh, Sahar Ghaffari Khaligh

**Affiliations:** 1 Department of Medical Physics, Faculty of Medical Sciences, Tarbiat Modares University, Tehran, Iran; 2 Skin and Stem Cells Research Center, Tehran University of Medical Science, Tehran, Iran; 3 Department of Technology of Radiology, Allied Medical Faculty, Shahid Beheshti University of Medical Science, Tehran, Iran; 4 Department of Pathology, Faculty of Veterinary Medicine, Semnan University, Semnan, Iran

**Keywords:** Biophysics, Acoustics, Oncology, Stem cells, Mesenchymal stem cells, Biological physics

## Abstract

Mesenchymal stem cells are mechano-sensitive cells with the potential to restore the function of damaged tissues. Low-intensity ultrasound has been increasingly considered as a bioactive therapeutic apparatus. Optimizing transplantation conditions is a critical aim for radiation-induced skin tissue injury. Therefore, the therapeutic function of adipose-derived mesenchymal stem cells to ultrasound stimulus was examined based on the mechanical index (MI). Mesenchymal stem cells were isolated from the adipose tissues of mature guinea pigs. An ultrasound system (US) was constructed with a 40 kHz frequency. The radiation-induced skin injury model was produced on the abdominal skin of guinea pigs by 60 Gy of radiation. Then, they were divided to 7 groups (n = 42): control, sham, US (MI = 0.7), AdMSCs injection, US AdMSCs (AdMSCs, under US with MI = 0.2), AdMSCs + US (AdMSCs transplantation and US with MI = 0.7) and US AdMSCs + US (combining the last two groups). The homing of stem cells was verified with fluorescence imaging. The groups were followed with serial photography, ultrasound imaging, tensiometry, and histology. The thickness of the skin was analyzed. Functional changes in skin tissue were evaluated with Young’s modulus (kPa). One-way ANOVA tests were performed to analyze differences between treatment protocols (p < 0.05). The results of Kumar’s score showed that radiation injury was significantly lower in the treatment groups of US AdMSCs and US AdMSCs + US than other groups after 14 days (p < 0.05). There was a significant difference in skin thickness between treatment groups with control, sham, and US groups after 60 Gy radiation and were closer to the thickness of healthy skin. Young’s modulus in US AdMSCs + US, US AdMSCs, and AdMSCs + US groups demonstrated a significant difference with the other groups (p < 0.05). Young’s modulus in US AdMSCs + US and US AdMSCs treatment groups were closer to Young’s modulus of the healthy skin. The histological results confirmed the improvement of acute radiation damage in the combined treatment method, especially in US AdMSCs + US and US AdMSCs groups with increasing the epithelialization and formation of collagen. An ultrasonic treatment plan based on a mechanical index of the target medium could be used to enhance stem cell therapy.

## Introduction

The majority of studies confirm that non-ionization and non-invasive methods with cell-therapy would affect skin tissue injury. Radiation-induced skin tissue injury, as the most common side effect of radiotherapy methods, interventional radiation therapy, and nuclear events, is being increased with new methods^1^. Reports indicate that ~ 95% of people who receive radiation therapy have experienced acute or chronic skin tissue radiation side effects^2^. Radiation tissue injuries at high doses or in combination with invasive procedures such as topical surgery can have deleterious effects on the epidermis, vascular structures, and dermis tissue^3^.

Radiation-induced tissue injuries are caused by DNA damage to skin cells, apoptosis, and decreased production of differentiated and proliferative factors such as fibroblast growth factor (FGF) and other gene expressions^4^. Ionizing radiation disrupts the cellular environment in the tissue. Radiation tissue injuries to the skin tissue include reduction of the regeneration process, drying of the skin tissue, loss of the epidermis, inhibition of angiogenesis, and skin ulceration and subcutaneous tissue injury^5^.

The idea of using interventional methods with allogeneic stem cells in skin damage is one of the most important issues in radiation-induced injury in regenerative medicine^6^. It has been observed that the presence of stem cells can increase the function and number of cells in the area affected by radiation injuries. Adipose-derived mesenchymal stem cells (AdMSCs) could help heal injured tissues by providing appropriate environmental conditions in the tissue by secreting growth factors. The presence, migration, proliferation, and differentiation of mesenchymal stem cells in the treated tissue may provide a promising therapeutic approach to improve radiation-induced skin tissue injury^7–9^.

Apart from the importance of secretory factors in cellular and intercellular processes, the use of biological or mechanical stimuli are very significant for regulation and acceleration of growth and differentiation, migration into injured tissues, and better utilization of allogeneic connectivity in therapeutic cell applications, especially stem cells^10,11^. Low-intensity ultrasound is a special type of energy that results in the transmission of acoustic pressure and mechanical stress. The main idea behind the use of low-intensity ultrasound in the medical field is the reconstruction of radiation injuries as a non-ionization method^12^.

Evidence indicates that integrins are candidates for sensing mechanical stimulation derived from the extracellular matrix. As a result, mechanical stimuli transmit local energy into the environment, supporting signal pathways for tissue and wound healing, which are a way to improve the efficiency and outcome of stem cell transplants^13^. There are a number of issues indicating that ultrasound can trigger several biochemical events at the cell surface and increase the secretion of growth factors and many anti-inflammatory molecules, reported by in vitro and in vivo studies^14^.

AdMSCs are also capable of receiving these mechanical stimuli before and after transplantation and can yield satisfactory results in injured tissues^15^. In the case of low-intensity ultrasound radiation, effective physical reasons along with biological and chemical reasons have to be considered. However, so far, no specific standard has been introduced for the therapeutic use of ultrasound, and a wide variety of radiation parameters have been utilized in studies^16^. Therefore, the effect of low-intensity ultrasound radiation based on the mechanical index parameter at the cavitation threshold on allogenic AdMSCs in the irradiated medium before and after transplantation was examined in this study.

Furthermore, it is necessary to examine the function of the ultrasound to be able to approach the therapeutic goals and reach an ideal knowledge of ultrasound function. The mechanical index represents the threshold for acoustic cavitation^17–19^. Since ionizing radiation delays the production of inflammatory cytokines in the skin tissue and reduces the healing period of damage, in this study, stem cells were used along with ultrasound waves to enhance the cumulative effect of the therapy procedure. The effect of ultrasound interaction on AdMSCs before (in vitro) and after transplantation (in vivo) in the skin was investigated in order to establish an effective stimulated mechanism for the regeneration of damaged tissue caused by radiation therapy.

## Results

### Animal model

**Figure 1:**
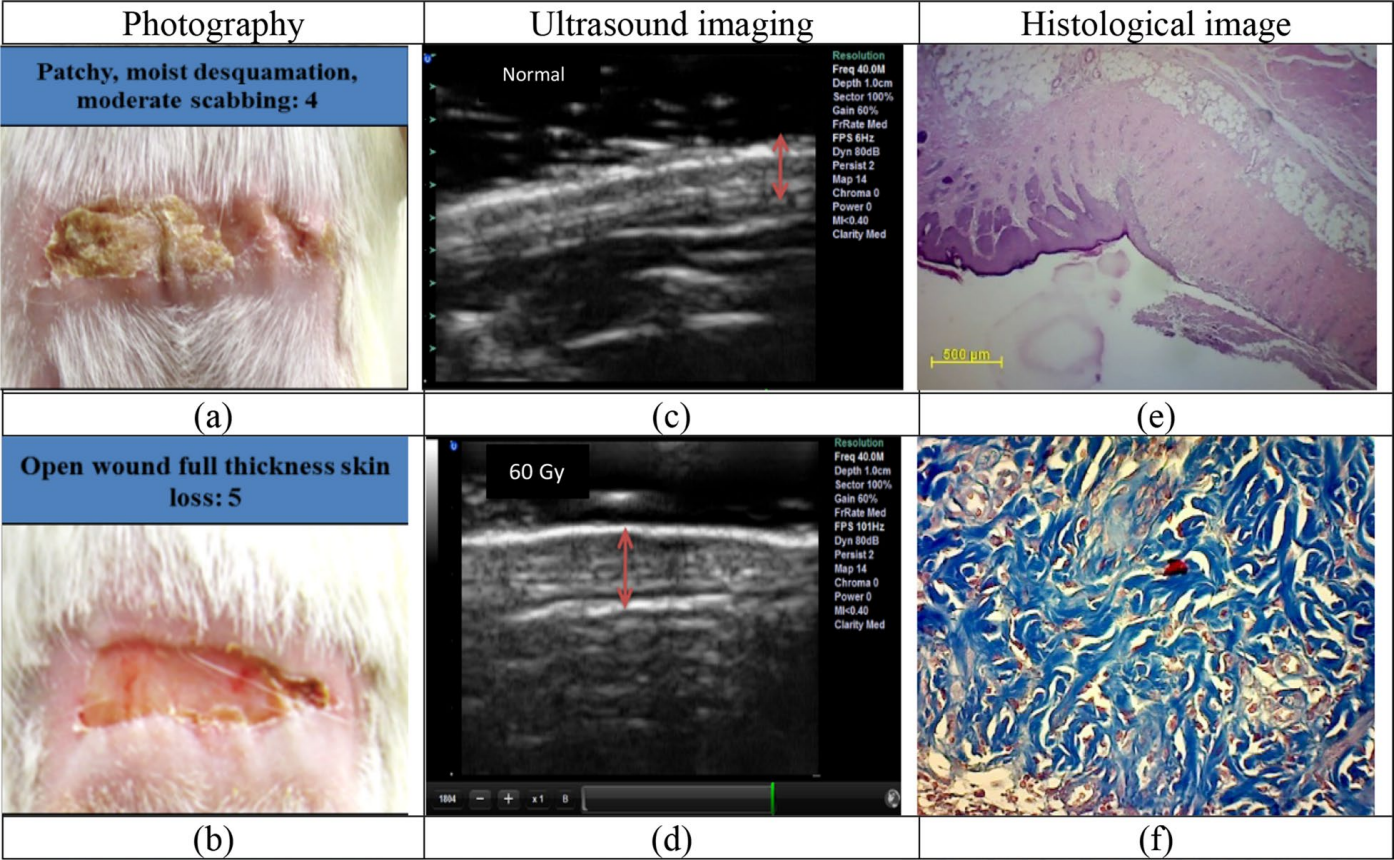
A sample of radiation-induced skin injury with 60 Gy of radiation: (**a**) photography of moist desquamation, and (**b**) the open wound, (**c**) ultrasound imaging of the normal tissue and (**d**) 60 Gy of radiation. Histological images of skin tissue samples in 60 Gy, (**e**) hematoxylin and Eosin (× 100), and (**f**) Masson’s trichrome (× 200).

Following the radiation-induced injury model, five additional guinea pigs received a radiation dose of 60 Gy. Neither internal organ injury nor any systemic side effects was observed in the model. Radiation-induced skin injury was evaluated with photographic images with the grading of radiation damage and thickness changes of skin layers, including epidermis and dermis, by ultrasound imaging and histological changes in Fig. 1.

Based on photographic images, during 17 days, open wounds appeared with a 60 Gy dose in the guinea pig abdominal skin tissue model (Fig. 1b). By day 5, all radiated areas displayed the acute effects of moderate erythema, mildly dry skin, and hair loss. By day 10, dry desquamation emerged in the area of a severe reaction. On day 14, acute radiation injury with moist desquamation was formed (Fig. 1a). The 40 MHz ultrasound images clearly show changes in the dermal layers and epidermis of guinea pig skin tissue in the model of acute radiation injury (Fig. 1d) compared to normal skin tissue (Fig. 1c). Most thickenings were detected by high-frequency ultrasonography in the layer of the dermis after 10 days (Fig. 1d). To confirm the wound grade, histological studies were applied 10 days after 60 Gy radiation. Skin tissue staining indicated acute radiation-induced injury with 60 Gy (Fig. 1e,f). The thickness of the skin layers increased due to the destruction of collagen fibers, as well as the adhesion and accumulation of non-functional fibers. Hematoxylin staining indicated increased inflammatory cells (Fig. 1e).

In Masson’s Trichrome staining, very thick collagen fibers with a noticeable density were observed in the dermis layer. The loss of hair follicles and vasculatures of the irradiated area was also seen in histological images. Due to the difference in images between the normal skin tissue and the skin tissue radiated with a 60 Gy dose, this radiation dose was selected as the target dose in the acute skin tissue radiation-induced injury model. Thus, therapeutic methods that influence the process of cell activity to prevent the progression or improve cellular and tissue injury could be evaluated in this animal model.

### AdMSCs

**Figure 2:**
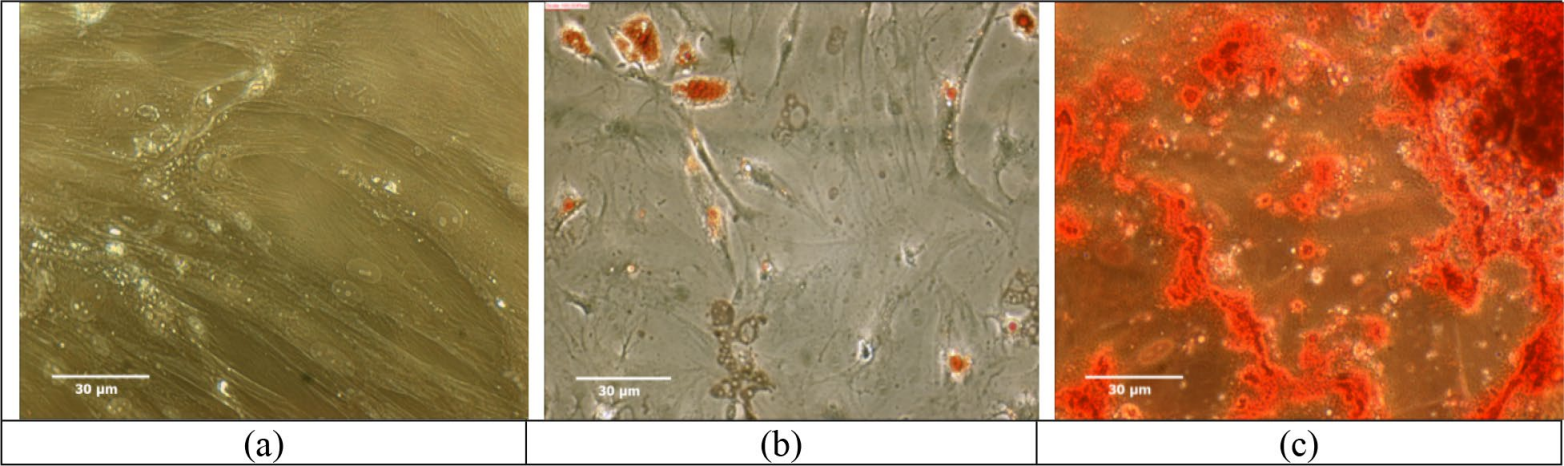
Differentiation potential of ADMSCs by the inverted microscope (× 400). (**a**) The negative control group, (**b**) adipogenic differentiation (Oil red staining), (**c**) osteogenic differentiation (Alizarin red staining).

In the enzymatic isolation method, AdMSCs were observed 24 h after cell culture. In the tissue explant method, AdMSCs were appearance and grown from the tissue explant after 7 days. These cells attach to the bottom of the plate. The morphology of AdMSCs had a spindle cell form and was similar to fibroblast cells (Fig. 2a). After 3 weeks, the differentiated cells into adipose cells were confirmed by oil red staining for the presence of intracellular lipid vacuoles. The deposition of calcium and mineral deposits of the extracellular matrix of differentiated osteogenic cells has been confirmed by Alizarin red staining. Adipogenic differentiation was verified by the detection of intracellular lipid vacuoles, apparent as intracellular red droplets (Oil red staining) (Fig. 2b). Osteogenic differentiation was verified by the detection of extracellular calcium deposition indicated by red nodules (Alizarin red staining) (Fig. 2c).

### Stem cells tracking

**Figure 3:**
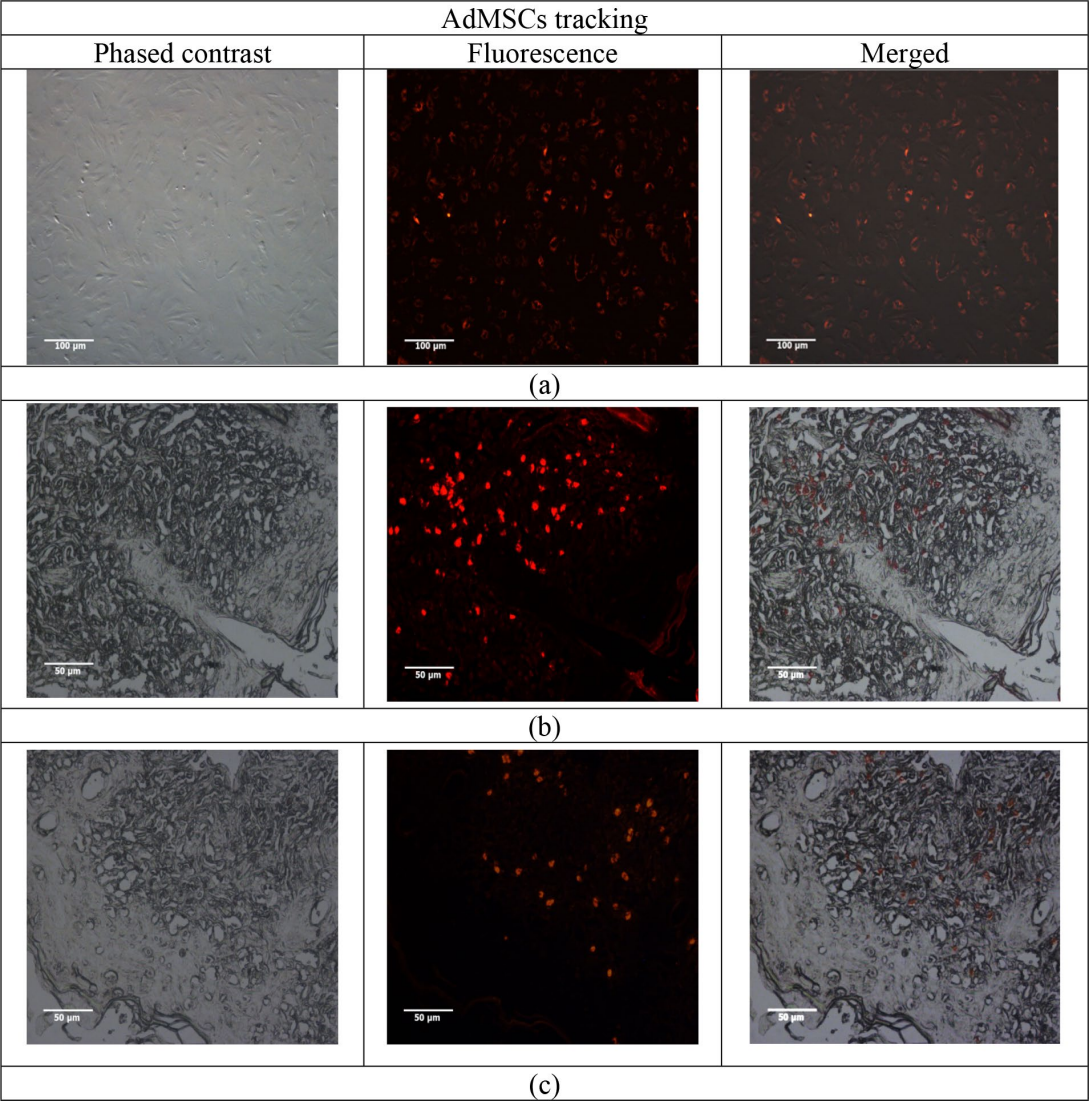
Characterization of guinea pig DiI-labeled AdMSCs by fluorescent microscopy, Phase contrast image of adherent and fibroblast morphology, DiI-fluorescence and merged image of optical and fluorescence images: (**a**) DiI-labeled AdMSCs in vitro (× 100), (**b**) Engrafted DiI-labeled AdMSCs were expressed within a dermal layer after intradermal transplantation into the tissue with radiation reactions after 7 days and (**c**) after 21 days (× 200).

AdMSCs were traced on the 7th and 21st days after intradermal transplantation via DiI staining. Especially, DiI_labeled AdMSCs in Fig. 3a were transplanted intradermally in the radiation field area to explore in vivo migration and traceability (Fig. 3b,c). AdMSCs were cultured to the 3rd passage before transplantation. All AdMSCs were indicated DiI-positive 24 h after labeling (Fig. 3a). To identify the survival, distribution, and differentiation characteristics of AdMSCs after transplantation, DiI-labeled AdMSCs (2 × 10^6^) were injected into radiation-induced skin injury and analyzed for cell presence three weeks later. In skin tissue sections, DiI-labeled AdMSCs were clearly distinguished in the tissue injury after 7 days (Fig. 3b). Radiation-induced damage was observed as dry desquamation with a score of 2.5 on the 7th day.

Cell tracking also showed that AdMSCs survived 21 days after transplantation. Wound healing of the damaged tissue was processed in 21st days. Microscopic analysis of the skin section provided evidence for the migration and homing of AdMSCs to the layers of the skin tissue. However, compared with the intense signal in 7 days at the radiation injury site, only a minimal number of the cells remained and bioluminescence was decreased 21 days after transplantation (Fig. 3c).

**Figure 4:**
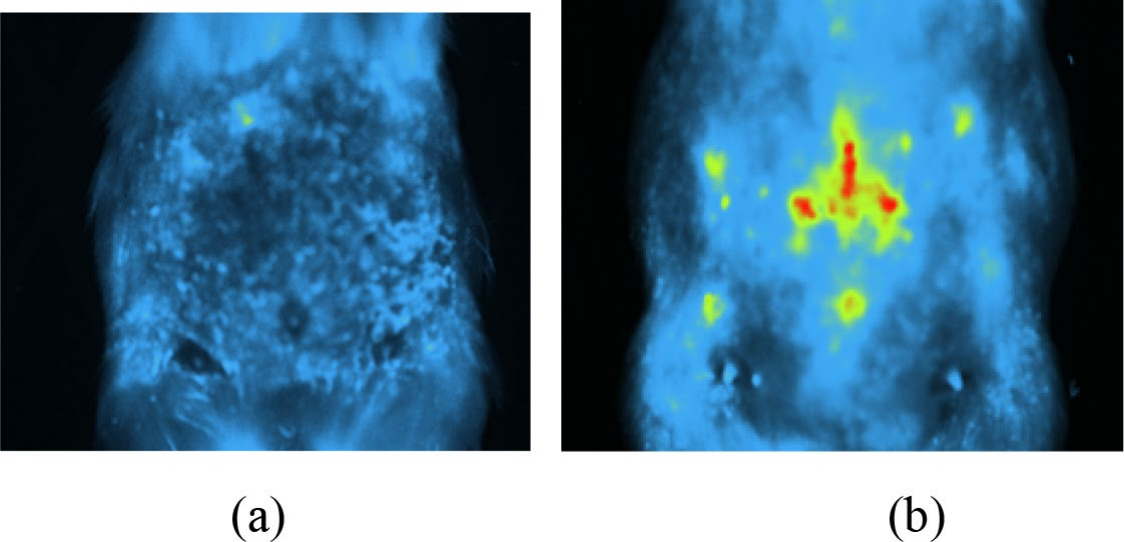
In vivo bioluminescent imaging of the guinea pig with radiation-induced skin injury following intradermal transplantation after 7 days: (**a**) AdMSCs transplantation, (**b**) DiI-AdMSCs transplantation.

In vivo imaging was performed 7 days after injections to determine the sporadic staining migration or diffusion of the DiI-labeled AdMSCs transplantation (Fig. 4).

There was no luminescence signal in Fig. 4a based on in vivo imaging due to the injection of AdMSCs without DiI staining. However, the luminescence signal was observed, depicting the migration and homing of DiI-labeled AdMSCs at the initial injection sites (center and edges). In the area of the radiation field, a localized signal was captured and represented real illumination from the injected cells (Fig. 4b).

### Mechanical index parameters in vitro and in vivo

**Figure 5:**
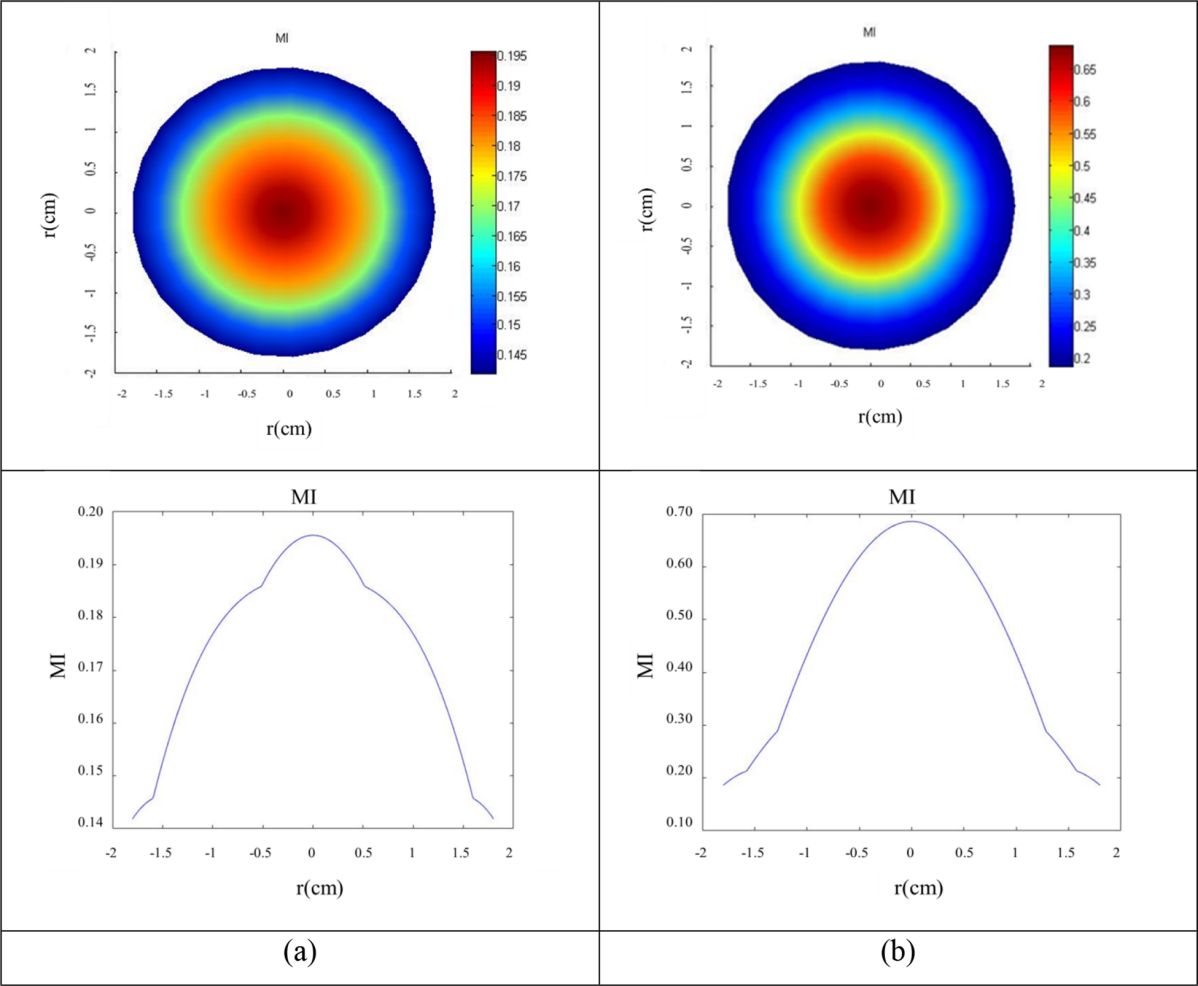
Radial contours of 40 kHz sonication with 0.23 W/cm^2^. (**a**) Threshold mechanical index in a 37 °C water environment at a 1.5 cm distance from the ultrasonic transducer, (**b**) threshold mechanical index in a 35–37 °C skin tissue at a distance of 0.1–0.5 cm. The color map represents the mechanical index range. Color change from blue to red determines an increase in the mechanical index range.

The mechanical index was calculated at a frequency of 40 kHz with a 0.4 cm near field at a distance of 4 cm from the ultrasonic source. In the line of axial propagation of the ultrasonic waves, the mechanical index was determined in a space with a radius of 1.8 cm at different distances from the ultrasonic source in the diffusion medium. The maximum mechanical index at the axial distance from the converter was extracted as the mechanical index (Fig. 5). In Fig. 5, the radial curves of the ultrasonic source in the selected groups are plotted. The radius chosen for the curve is the target radius, i.e. the cell plate and the size of the radiation field. As the curves demonstrate, the mechanical index is higher in the center, and upon moving closer to the radiation environment, the mechanical index values decrease. Figure 5a,b display the mechanical index changes in water (MI = 0.20) at 0.50 cm and skin tissue (MI = 0.70) at 0.10 to 0.50 cm from the ultrasonic source.

After determining the exposure parameters by the mechanical index model, such as frequency, intensity, and distance parameters, the exposure time was obtained by the thermal control protocol. The low-intensity ultrasound irradiation time was obtained 140.00 ± 0.01 s in the culture medium and 176.00 ± 0.05 s in skin texture.

### Evaluation of combined treatment

**Figure 6:**
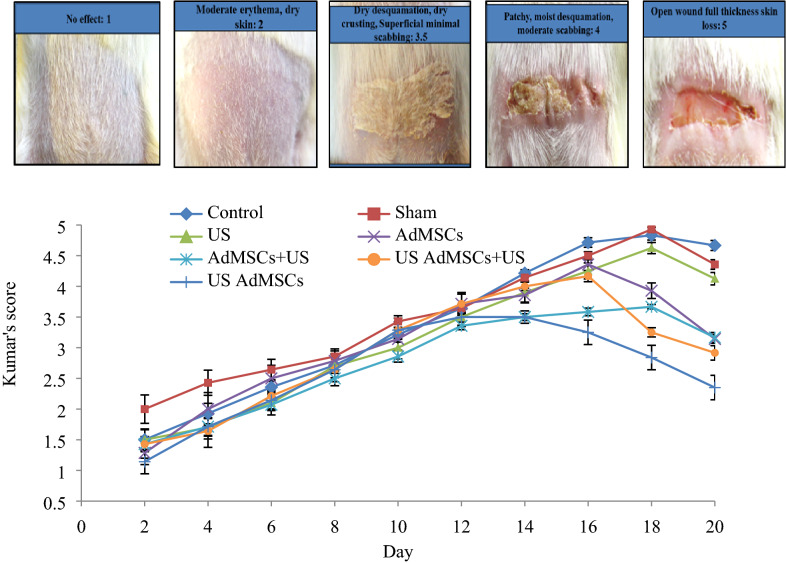
Score changes of acute radiation damage to the skin tissue (1–5) on the 2nd to 20th day after 60 Gy radiation in different groups (control, sham; treated with normal saline injection, US; sonication 24 h after 60 Gy radiation, AdMSCs: treated with AdMSCs, US AdMSCs; treated with AdMSCs and sonication with 0.2 MI—24 h before transplantation, AdMSCs + US; treated with AdMSCs transplantation and sonication with 0.70 MI—24 h after transplantation, US AdMSCs + US group; AdMSCs transplant under sonication 24 h before (0.2 MI) and 24 h after (0.7 MI) transplantation) based on Kumar’s scales.

To prevent the progression of acute radiation damage a combined treatment method was performed by cell therapy and mechanical stimuli. Radiation-induced injury scores of the skin tissue are presented in Fig. 6 based on Kumar’s study.

The results of Fig. 6 indicate the changes in skin tissue caused by 60 Gy radiation and the effect of combined treatment on the acute radiation damage of the skin tissue. In the first week, the changes were in the form of dry desquamation with a grade of 2 to 2.5, and in the second week (14 days after 60 Gy irradiation), the changes were in the form of moist desquamation of 3.5 to 4. Statistical analysis revealed that there was no significant difference between the treatment groups with control, sham, and US groups from 2nd to 14th days. Nevertheless, radiation injury was significantly lower in the treatment groups (US AdMSCs and AdMSCs + US). From the 14th to18th days, a significant difference was observed between the US AdMSCs and US AdMSCs + US with control, sham, and US groups (p < 0.05). The US AdMSCs group showed improvement in skin tissue 20 days after 60 Gy radiation; the statistical analysis showed a significant difference with other groups (p < 0.05) (Fig. 6).

**Figure 7:**
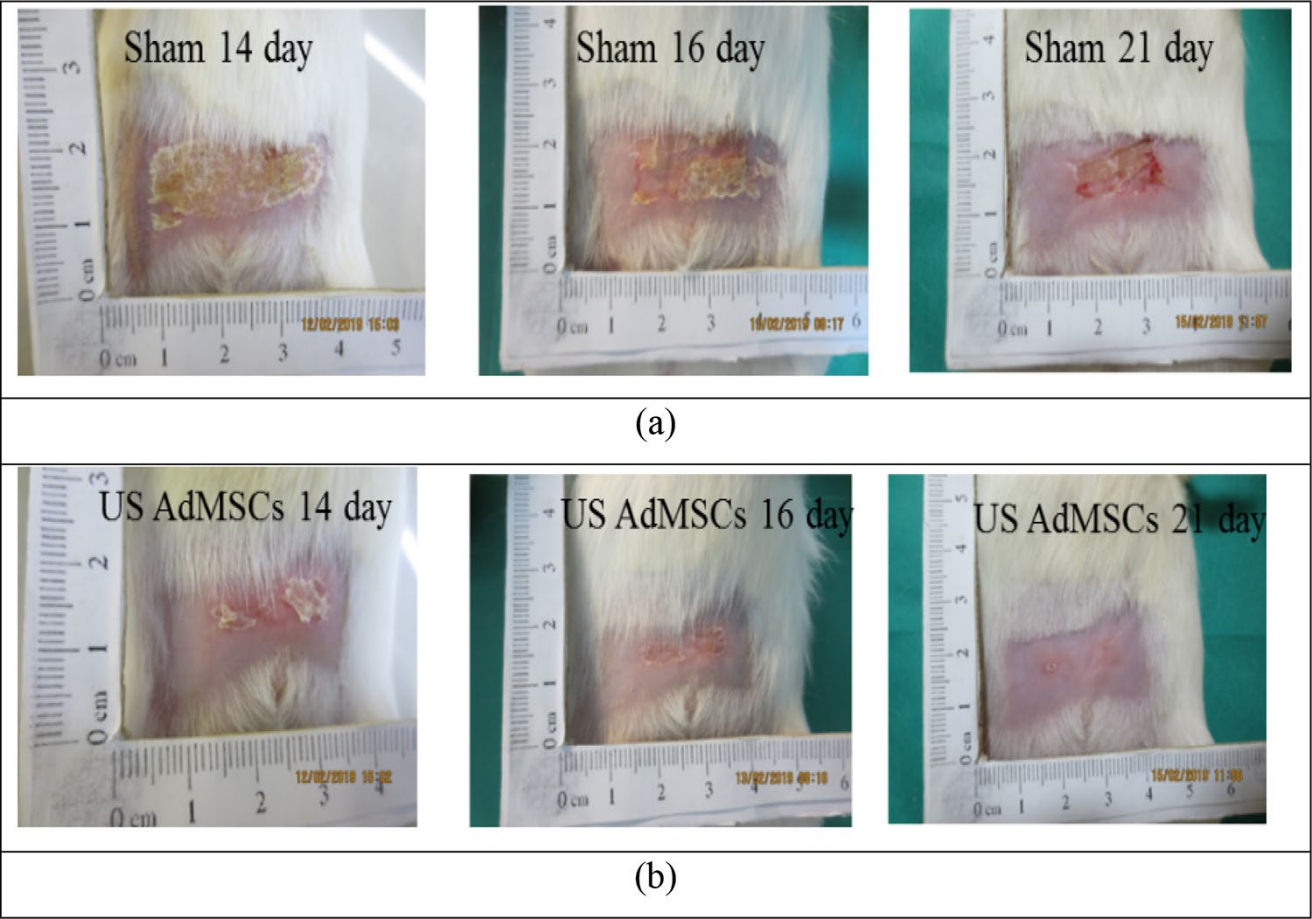
Acute radiation injury to the skin tissue within 21 days, (**a**) sham group, (**b**) US AdMSCs group.

Figure 7 shows the effect of the stem cell therapy protocol and low-intensity ultrasound waves on the recovery of acute skin tissue damage caused by radiation therapy compared to the sham group on days 14, 16, and 18.

Table 1 presents the evaluation of skin tissue thickness after 7 days (onset of dry desquamation) and 14 days (onset of moist desquamation) of 60 Gy irradiation. The thickness of the skin tissue comprises the epidermis and the dermis layers and the total thickness in the treatment, control and sham groups.

**Table 1:** Mean (SD) of skin tissue thickness (epidermis, dermis, and total thickness) during the treatment process in different groups after 60 Gy radiation.

Layer	Day	Control	Sham	US	AdMSCs	US AdMSCs + Us	US AdMSCs	AdMSCs + US	Normal
Epidermis	7	0.35 (0.04)	0.40 (0.06)	0.40 (0.05)	0.26 (0.04)	0.18 (0.02)	0.17 (0.03)	0.20 (0.03)	0.13 (0.05)
	14	0.43 (0.08)	0.43 (0.08)	0.42 (0.06)	0.25 (0.04)	0.17 (0.03)	0.16 (0.02)	0.18 (0.03)	0.13 (0.05)
Dermis	7	1.41 (0.13)	1.82 (0.19)	1.50 (0.08)	1.27 (0.07)	1.20 (0.17)	1.03 (0.08)	1.21 (0.09)	0.73 (0.03)
	14	1.50 (0.16)	1.69 (0.09)	1.54 (0.10)	1.31 (0.10)	1.28 (0.15)	0.99 (0.06)	1.15 (0.11)	0.73 (0.03)
Total	7	1.79 (0.12)	1.89 (0.10)	1.74 (0.08)	1.63 (0.07)	1.27 (0.09)	1.20 (0.08)	1.50 (0.08)	0.89 (0.05)
	14	1.86 (0.15)	1.96 (0.07)	1.66 (0.09)	1.60 (0.09)	1.40 (0.07)	1.25 (0.08)	1.50 (0.10)	0.89 (0.05)

There was a significant difference in skin thickness between treatment groups and control, sham, and US groups in the 1st and 2nd week after 60 Gy radiation. The lowest skin thickness was measured in the US AdMSCs group (1.25 ± 0.08 mm) with the dermis layer of 0.99 ± 0.06 mm and the epidermis layer of 0.16 ± 0.02 mm. The thickness of normal guinea pig skin tissue was 0.89 ± 0.04 mm, with the dermis layer of 0.73 ± 0.03 mm and the epidermis layer of 0.13 ± 0.05 mm. The US AdMSCs group showed a significant difference after 7 and 14 days of 60 Gy radiation compared to other groups (p < 0.05). The highest skin thickness was obtained in the sham group (1.92 ± 0.07 mm) with the dermis layer of 1.69 ± 0.05 mm and the epidermis layer of 0.42 ± 0.06 mm. The results show that the thickness of the skin in the treatment groups is closer to the thickness of healthy skin (Table 1).

**Figure 8:**
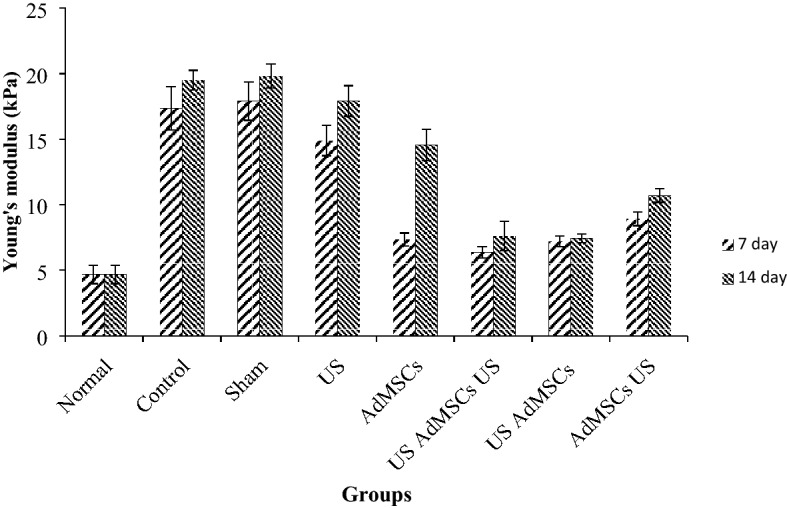
Mean (SD) of Young’s modulus (kPa) during the treatment process 7 and 14 days after 60 Gy radiation compared to normal tissue with a 4.68 ± 0.69 kPa Young’s modulus.

Since the change of skin elasticity is an important feature in distinguishing damaged tissue from healthy tissue, Young’s modulus (elasticity) of the skin tissue was calculated in different groups after stress was applied to the tissue. The results of Young’s modulus (kPa) are depicted in Fig. 8.

**Figure 9:**
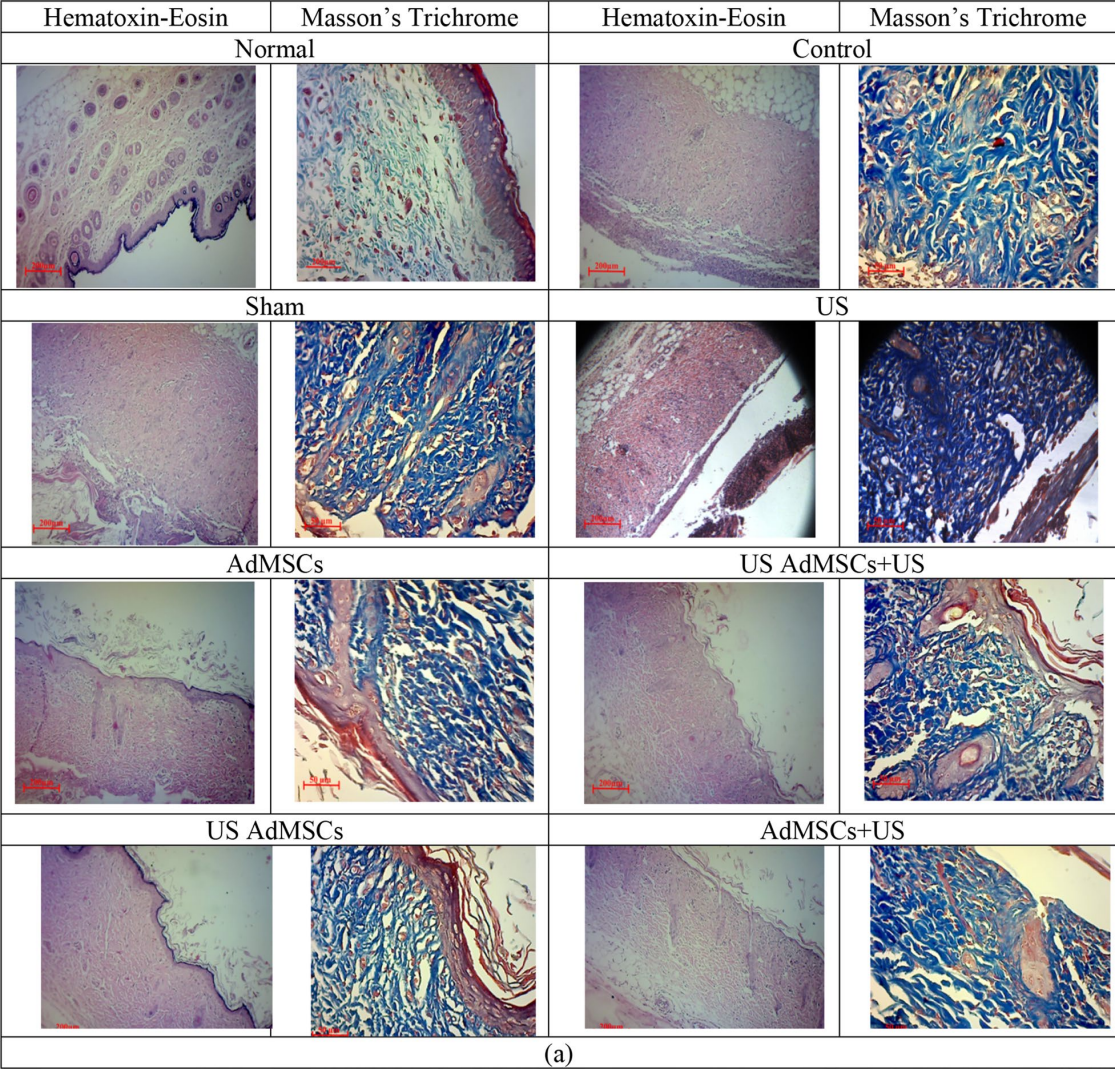

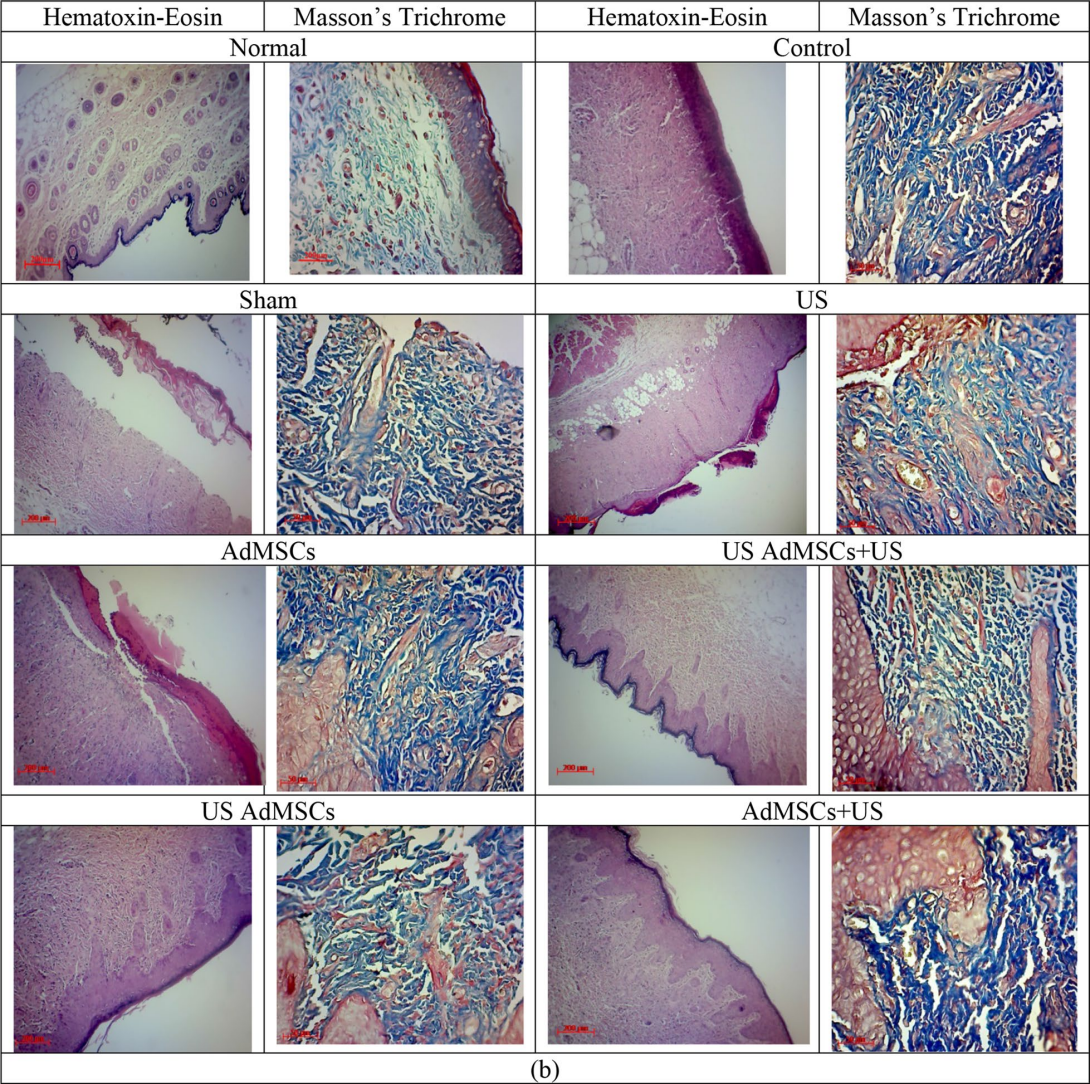
Histological images of the skin tissue in control, sham, 40 kHz US, AdMSCs, US AdMSCs + US, US AdMSCs, and AdMSCs + US groups and normal skin with Hematoxin-Eosin staining (× 100) and Masson’s trichrome staining (× 200); (**a**) 14 days and (**b**) 21 days after treatment.

The trend of Young’s modulus in the US AdMSCs + US treatment group with 7.56 ± 1.12 kPa and US AdMSCs group with 7.44 ± 0.33 kPa showed a significant decrease in skin elasticity compared to the control (19.56 ± 0.74 kPa) and sham group (19.79 ± 0.91 kPa) (p < 0.05). Also, Young’s modulus in US AdMSCs + US and US AdMSCs treatment groups displayed a significant difference with the AdMSCs group (14.56 ± 1.2 kPa) 14 days after 60 Gy radiation (p < 0.05). The results indicate that Young’s modulus in US AdMSCs + US and US AdMSCs treatment groups is closer to the Young’s modulus of the healthy skin (4.68 ± 0.69 kPa). The hardening of the skin tissue, biochemical changes, destruction of collagen elastic fibers, and cell apoptosis of the skin tissue can be the reasons for an increase in the hardness of skin tissue (Fig. 8). Histological images are depicted in Fig. 9a after 14 days.

At the end of the treatment, the skin tissue of the treatment region was harvested, and biomechanical parameters were extracted by the tensiometry method in all treatment groups (Table 2). The skin biomechanical parameters included the skin stiffness (N/mm), the tolerable maximum force required for tissue rupture (R_m_), the maximum displacement of the skin tissue in the tolerable maximum force (_ɛFmax_), and the energy absorbed at the point of maximum strength (W_up to Fmax_) which assessed the effectiveness of the treatment throughout the healing phase on the 21st day.

**Table 2:** Biomechanical parameters at the end of the treatment process 21 days after a 60 Gy dose compared to the normal tissue.

Parameter	Control	Sham	US	AdMSCs	US AdMSCs + US	US AdMSCs	AdMSCs + US	Normal
Stiffness (N/mm)	1.10 (0.15)	1.01 (0.12)	1.03 (0.15)	0.80 (0.09)	0.66 (0.04)	0.59 (0.04)	0.69 (0.04)	0.52 (0.05)
R_m_ (N)	16.83 (1.90)	19.65 (4.55)	19.43 (4.88)	10.86 (6.44)	7.98 (2.66)	12.95 (1.98)	10.85 (5.73)	5.11 (0.87)
ɛF_max_ (mm)	16.81 (3.84)	21.28 (4.81)	19.00 (5.87)	13.86 (5.60)	11.66 (5.94)	12.35 (1.89)	14.50 (7.04)	9.74 (1.15)
W_Fmax_ (N mm)	173.28 (8.88)	140.82 (5.62)	146.71 (3.71)	26.61 (4.67)	29.46 (3.95)	45.34 (6.21)	36.50 (9.62)	31.14 (4.58)

Table 2 shows a significant difference between stiffness in the normal skin with that of control, sham, and US groups (p < 0.05). Results showed that the stiffness values in treatment groups of US AdMSCs + US, US AdMSCs, and AdMSCs + US groups do not significantly differ from the normal (p > 0.05), indicating skin tissue repair in stem cell treatment groups. The tolerable maximum force required for skin tissue rupture showed a significant difference between normal skin, US AdMSCs + Us, US AdMSCs, and AdMSCs + US groups with control, sham, and US groups (p < 0.05). Based on the maximum displacement of the skin tissue in the tolerable maximum force (ɛF_max_), the results have improved in treatment groups, including US AdMSCs + Us and US AdMSCs (p < 0.05). The energy absorbed at the point of maximum strength (W_up to Fmax_) demonstrated a significant difference between AdMSCs, US AdMSCs + US, US AdMSCs, and AdMSCs + US groups with control, sham, and US groups (p < 0.05). Moreover, the energy absorbed in the US AdMSCs + US group was similar to the normal skin tissue.

In Fig. 9, the histological images of skin tissue samples with Hematoxin-Eosin staining and Masson’s trichrome staining are depicted in the control, sham, 40 kHz US (0.7 MI, 24 h after 60 Gy radiation), AdMSCs (transplantation), US AdMSCs (sonication of AdMSCs with 0.2 MI, 24 h before transplantation), AdMSCs + US (AdMSCs transplantation and skin sonication with 0.70 MI, 24 h after transplantation), US AdMSCs + US (AdMSCs transplant under sonication with 0.2 MI 24 h before, and skin sonication with 0.7 MI 24 h after transplantation), and normal skin, 14 and 21 days after treatment. Histological images 21 days after 60 Gy irradiation in different treatment groups well illustrate the structural changes of the tissue at the end of the treatment.

In the histological images of the healthy skin, the layers of epidermis and dermis are visible without inflammation. Basal cells and keratinocytes are seen in the epidermis layer. The presence of fibroblast cells and collagen fibers in the dermis is remarkable. However, in the control and sham samples after 14 days of 60 Gy radiation, inflammatory mononuclear cells with inflammation are observed in the dermis layer. The collagen strands are dense, intertwined, and non-homogeneous. In the skin tissue of the US group and AdMSCs + US group, edema in the dermis layer and presence of macrophage and mononuclear inflammatory cells are observed in the radiated area. In the AdMSCs group, cells of the epidermis layer are seen. The dermis layer also contains fibroblast cells with edema, and the presence of restricted mononuclear inflammatory cells is evident. Thick collagen fibers in the dermis can also be observed. The histological sample of the US AdMSCs + US group and the US AdMSCs represent cell layers in the epidermis. Nevertheless, in the tissue sample of the US AdMSCs group, there is a complete epidermis layer. The collagen strands in the dermis are also perfectly parallel, with very few mononuclear inflammatory cells observable. The presence of fibroblast-like spindle cells in the dermis is also visible. In this group, the progress of radiation reactions is well delayed, and the degree of tissue and cell injury is reduced after 14 days (Fig. 9a).

At the end of the treatment, in the 60 Gy-radiated region in the control, sham, US, and AdMSCS samples, epithelialization was not completely formed after 21 days (Fig. 9b). Due to the formation of newly formed vessels, the presence of fibroblast cells and the presence of mismatched collagen filaments in the dermal area suggest repair in this area of skin is at the end of the broiler or granulation tissue stage. The presence of inflammatory cells, which are mostly lymphocytes and macrophages, is scarce in this area. In the combined treatment groups, such as US AdMSCs + US, US AdMSCs, and AdMSCs + US, the epithelialization and formation of the epidermis layer are fully performed after 21 days. A number of vessels are seen, and more fibroblast cells are found in the dermis. Thick and homogeneous collagen fibers are present in the dermis. The presence of mononuclear inflammatory cells also indicates the complete repair of the affected area.

Grade of wounds in terms of epithelialization, inflammatory cells, presence of fibroblasts, angiogenesis and collagen factors are shown in Table 3. The histological results confirm the amelioration of acute radiation damage in the combined treatment method in the mechanical index of the irradiated environment, especially in US AdMSCs + US and US AdMSCs groups (Fig. 9b, Table 3). Process of treatment, fibroblast concentration, epithelialization, angiogenesis and collagen formation in US AdMSCs + US and US AdMSCs groups were better than other groups and closer to healthy skin.

**Table 3:** Mean (SD) of grade of wounds in terms of epithelialization, inflammatory cells, presence of fibroblasts, angiogenesis and collagen during the treatment process in different groups.

Factors	day	Control	Sham	US	AdMSCs	US AdMSCs + Us	US AdMSCs	AdMSCs + US	Normal
Epithelization	14	3	3	3	1	**4**	**4**	**4**	**4**
	21	2	2	2	1	**4**	**4**	**4**	**4**
Inflammatory cells	14	2	2	3	2	**1**	**1**	3	**0**
	21	3	3	2	3	2	**1**	2	**0**
Fibroblasts	14	2	2	2	2	**1**	**1**	2	**0**
	21	3	3	3	3	**1**	**1**	2	**0**
Angiogenesis	14	3	2	2	3	2	**1**	2	**0**
	21	2	2	2	3	**1**	**1**	2	**0**
Collagen	14	2	2	2	2	2	**3**	2	**4**
	21	3	2	2	2	**4**	**4**	**4**	**4**

Bold font represent the values are close to the normal group

## Discussion

Recent studies have shown the effects of mechanical vibrations, including low-intensity ultrasound, as mechanical stimuli required for stem cell proliferation and differentiation. AdMSCs are capable of receiving mechanical stimulation, and ultrasound can increase the expression and differentiation of mesenchymal cell growth factors^11,20,21^.

In 1994, the Food and Drug Administration approved low-intensity ultrasound as a safe and effective treatment for bone fractures. So far, however, no specific standard has been introduced for the therapeutic use of low-intensity ultrasound^22^. The present study attempted to introduce the usage standard of ultrasound based on the cavitation interaction on the threshold of acoustic cavitation of various therapeutic environments such as culture medium and skin tissue.

Acoustic cavitation is a major interaction of low-frequency waves in causing structural changes in skin tissue^23^. In the combined treatment, the effects of ultrasound on cells before and after the transplantation of AdMSCs at the cavitation threshold mechanical index of the culture medium and skin tissue were studied for delaying and preventing radiation damage. In a similar study, Ling et al.^12^ investigated the effect of ultrasound radiation at a frequency of 0.25 MHz and an intensity of 30 mW/cm^2^ on AdMSCs before transplantation. After 5 days of ultrasonic irradiation for 30 min, the cells were injected through the tail vein of a mouse which had ovarian failure due to chemotherapy. At the end of the study, the results showed that the rate of inflammation and apoptosis in the bladder tissue treated with cells irradiated with ultrasound was improved, and the bladder function was also improved compared to AdMSCs alone. Sonication of cells before transplantation results in better utilization of the stem cell transplantation method.

Also, Mann^24^ studied the effect of low-frequency ultrasound (45 kHz) with 15 and 30 mW/cm^2^ on fibroblast and osteoblasts cells. The results revealed that the proliferation of cells was increased by the synthesis of collagen proteins and growth factors. Therefore, ultrasound radiation on cells before transplantation results in better utilization of the stem cell transplantation method.

Kinoshita et al. ^25^ reported an increase in the thickness of the epidermis and dermis layers in the irradiated groups in the 1st and 2nd weeks. High-frequency ultrasonography evaluation showed that the US AdMSCs group had a total skin thickness of 1.20 ± 0.08 mm, the dermis layer of 0.99 ± 0.06 mm, and the epidermis layer of 0.16 ± 0.02 mm, and thus had the closest skin tissue thickness to the normal skin in the treated groups (Table 1). The presence of keratinocyte cells in the dermis layer and fibroblast cells in the dermis layer showed a decrease in cell apoptosis and an increase in tissue function. Ultrasound in acoustic cavitation threshold with 0.20 mechanical index could be impacted in the expression and secretion of cell growth factors in culture conditions before transplantation. To evaluate skin tissue, the Young’s modulus of US AdMSCs + US treatment group (7.56 ± 1.12 kPa) and US AdMSC group (7.44 ± 0.33 kPa) was nearest to the Young's modulus of the normal skin group (4.68 ± 0.69 kPa). In addition, the Young’s modulus of US AdMSCs + US and US AdMSCs treatment groups showed a significant difference with the AdMSCs group (14.56 ± 1.20 kPa) (p < 0.05) (Fig. 8).

So, acoustic cavitation threshold interaction can affect cells and the environment. Optimal conditions are created by mechanical index modeling for healing the injury in the first days. The bubbles move around the equilibrium radius at a stable state in the mechanical index parameter. Through integrins that act as mechanoreceptors, these vibrations transfer into the cell and promote the attachment of different focal adhesion adaptor proteins to increase proliferation and gene expression^26^. Typically, the activation of an Extracellular-signal-Regulated Kinase (ERK) signaling pathway, which is used to mediate cell division, migration, and survival, is stimulated by a mixture of extracellular chemicals and environmental stresses^25^. The permeability of the cell membrane to calcium and sodium ions changes by acoustic cavitation, thus increasing protein synthesis in cells^27^.

In the present study, the difference between Young’s modulus of the control and sham groups with treatment groups was well observed by non-invasive elastography in the 1st and 2nd weeks.

Thanik et al.^28^ used Young’s modulus to determine the rigidity of the skin tissue invasively. The results revealed changes in the elasticity of the irradiated skin tissue compared to the normal group.

Based on stiffness results, the skin tissue function was similar to normal tissue and was confirmed histologically in the combined stem cell and ultrasound treatment (Fig. 9). After 21 days of 60 Gy radiation, in treated US AdMSCs, US AdMSCs + US, and AdMSCs + US groups, complete epithelialization was observed. Moreover, complete epithelial layer formation and more vessels, fibroblast cells, and collagen fibers were observed in the dermis layer (Fig. 9). This evidence indicates the effect of sonication on the three phases of wound healing, i.e. inflammation, proliferation, and regeneration, in acute radiation damage to the skin tissue. In the control group, an increase in the thickness of the radiated skin tissue, collagen deposition, and a large number of inflammatory cells and, consequently, an increase in collagen accumulation, a decrease in elasticity, and a contraction of the skin tissue were observed. Lv et al.^29^ evaluated the combined effect of low-intensity ultrasound and pluripotent stem cells on mouse sciatica nerve recovery on a surgical site. The mechanical properties of the neural canal were studied. However, in this study, the physical reason for the choice of low-intensity ultrasound is not well determined in vivo.

Therefore, in the present study, the treatment design in different environments and a standard based on a mechanical index threshold were introduced. Also, in a 2017 study, spermatogonial cells were affected by the ultrasound mechanical index. The groups with a threshold mechanical index had the highest reproducibility^18^.

Obviously, this range of mechanical index is different for cell cultures and tissues and should have an acoustic arrangement. Ultrasound also leads to the migration of stem cells to the affected area. In 2009, Ghanem and et al. performed the stimulation of ultrasound at the target site on transplanted mesenchymal cells in the cardiac tissue after acute myocardial infarction. The results showed that the rate of mesenchymal stem cell migration reached its maximum after 60 min^30^. The presence and migration of cells into the damaged skin tissue on the 7th and 21st day were observed by fluorescence and animal imaging (Figs. 3, 4).

In this study, the mechanism of the effect of low-intensity ultrasound on the acoustic cavitation threshold based on a mechanical index equation showed the effect of this interaction on the improvement of stem cell function and acute radiation damage to the skin tissue. The low-intensity ultrasound generated acoustic pressure wave can transmit to cell membrane and cytoskeletal structures, causing a number of signaling pathways and transcription of genes^31^. Focal Adhesion Kinase (FAK) and Steroid co-activator (Src) are stimulated by mechanical ultrasound interactions. These factors play important functions in integrin-mediated signal transductions and a wide range of cellular responses.The growth factor or cytokine-induced integrin/mitogen-activated protein kinase (MAPK), integrin/Ras/MAPK/nucleus, and other signaling pathways mediate cell proliferation^14,32^.

Studies report that mechanical stimuli can facilitate wound healing by collagen synthesis, macrophage activation, and angiogenesis stimulation, and leads to increased proliferation of fibroblast cells, which is the most critical effect in tissue radiation injury, leading to cell death and necrosis in the tissue^9^.

Portas (2016) studied the therapeutic effect of mesenchymal stem cells on acute radiation damage to human skin tissue. After two years of stem cell treatment, wound healing improved with complete skin recovery^7^. Lataillade also examined a new approach to radiation injury and autologous mesenchymal stem cell treatment. No acute reaction was observed from stem cell infusion, and wound healing occurred 75 days after irradiation^33^. The results of the present study indicated that stem cell therapy is associated with a reduction and suppression of the inflammation process, improvement of blood supply, angiogenesis, and improvement of skin tissue in the affected site, whereas a combined modality is required to reduce recovery time.

Furthermore, in most studies, the progression of the radiation effects was delayed, and no progression of the complication was observed before dry desquamation in the treated groups. Also, the size of the wound was smaller, and the wound healed faster^34^. The results of this study also indicated that the degree of acute radiation damage in groups treated with AdMSCs was decreased. However, maximum decrease was observed in the Us AdMSCs groups, whereas in the control groups, open wounds were observed (Kumar’s score of 5) (Fig. 6). The bio-effect of mechanical stimuli on cellular functions such as proliferation and gene expression has created optimal conditions in sonicated cells before transplantation.

In terms of autologous and allogeneic transplants, Ricconono studied the effect of autologous and allogeneic bone marrow-derived stem cell transplants on skin radiation damage. Clinical signs of irradiated skin tissue were classified based on symptoms, such that, in the best case, it had a score of 2.40 in the allogeneic group, at the end of the study, which was similar to the control group. Clinical signs of irradiated skin tissue were classified based on symptoms, such that, in the best case, it had a score of 2.40 in the allogeneic group, at the end of the study, which was similar to the control group. The results indicated that topical transplantation can play an important role in the early stages of wound healing, and the effects of healing of multipotent skin cells are evident. For a better conclusion to be drawn, cell therapy with allogeneic transplantation was suggested with other noninvasive methods^35^.

Therefore, the improvement of allogeneic AdMSCs culture conditions with sonication in the acoustic cavitation threshold caused a significant decrease in wound healing. Histologically, the sonicated AdMSCs migrated to the area of skin lesions and directly participated in healing.

## Conclusion

A combined treatment of low-intensity ultrasound mechanical index as a parameter affecting the allogeneic adipose-derived mesenchymal stem cells could repair acute radiation injury and increased skin tissue function. It is concluded that the transplantation of AdMSCs sonicated with attention to the mechanical index exerts a significant effect on acute radiation injury before transplantation (in vitro) compared to after transplantation (in vivo). Based on the results of this study, an ultrasound treatment plan based on the mechanical index of the cavitation threshold of the target environment can be adopted for enhancing cell therapy.

## Materials and methods

The study design was approved by the medical ethics committee (IR.TMU.IEC.1395.541).

### Model of acute radiation injury of guinea pig skin tissue in radiation therapy

**Figure 10:**
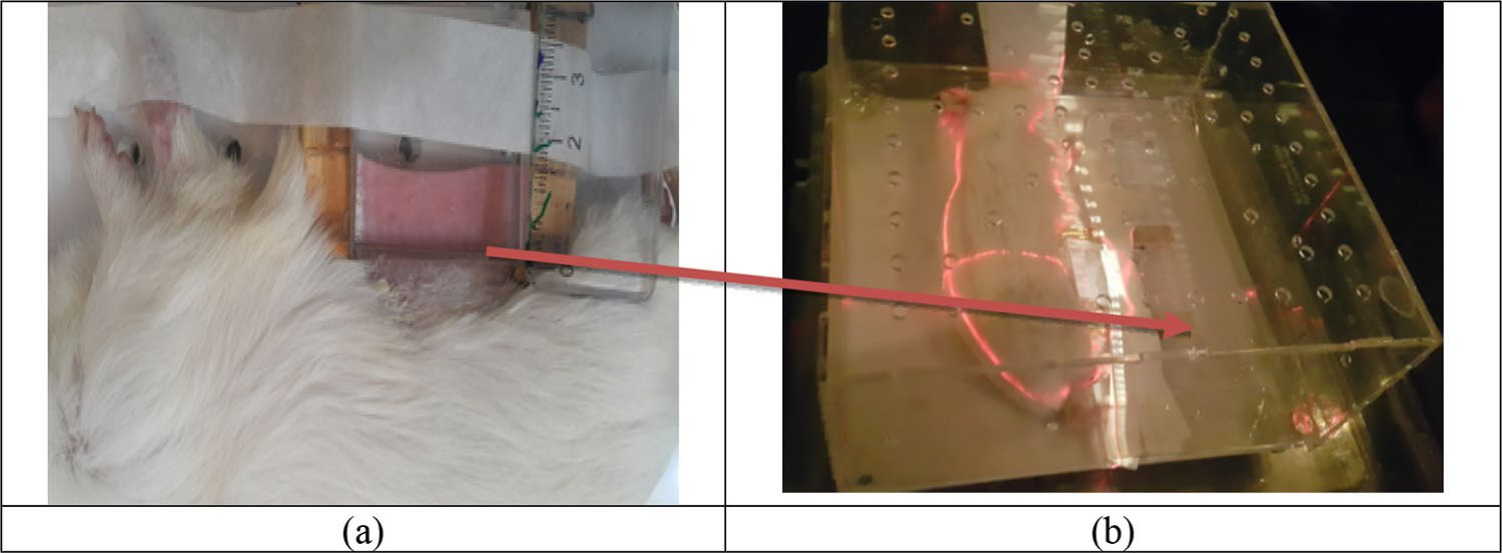
The skin distraction of the radiation-induced injury model: (**a**) under anesthesia and hair removal, the skin was held outward using a low-pressure wooden clamp, (**b**) the guinea pig was affixed to a plexiglass box and radiated with a linear accelerator radiotherapy apparatus (6 MV, Elekta Compact) with a dose of 60 Gy and dose rate of 2 Gy/min.

Mature female Guinea pigs (Hartly Dunkin, n = 5, 6 weeks old, 200–250 g) were selected and housed within the Laboratory Animal Center of Tarbiat Modares University (Tehran, Iran). In this model, due to the inability to dose fraction, the guinea pig skin tissue is being pulled with a clamp in the desired area to prevent injury to the internal organs (Fig. 10a). Guinea pigs euthanized by intraperitoneal injection of ketamine 10% (Alfasan, Woerden, Holland) and xylazine 2% (Alfasan, Woerden, Holland). They were also fixed in the plexiglass box for maintenance, transportation and hygiene at the hospital and were transferred to the linear accelerator room for radiation (Fig. 10b). Acute radiation injury to the skin tissue has been induced through a 6 MV linear acceleration radiotherapy (Elekta Compact, Au055, Stockholm, Sweden) with doses of 60 Gy, dose rates 2 Gy/min and a 7545 unit monitor at a distance of 100 cm SSD (Source to Skin Distance) and a 3 × 3 cm^2^ field size.

The combined treatment of ultrasound radiation and allogeneic transplantation of AdMSCs was studied on the acute radiation injury of guinea pig skin tissue in order to delay the time of injury and prevent its progression in the tissue. A single radiation dose was selected to cause acute skin injury of the type of moist desquamation (grade 4) and higher. This side effect is one of the most likely acute side effects and may result in the disruption of the epithelium and decrease the ability of the skin function^36^. The radiation-induced skin injury model after 60 Gy of radiation was confirmed by histological, photography, and ultrasound images, and its assessment was repeated five times.

### Clinical follow-up

During the follow-up period, photographic examination was performed 24 h after 60 Gy of radiation daily. The parameters related to the severity of skin injury and the type of injury were studied according to Kumar’s study criteria and those of other related articles. The radiation injury scores were 1 for the healthy condition (no difference with lateral healthy tissues, no effect); 1.5 for minimal erythema and dry skin; 2 for moderate erythema and dry skin; 2.5 for marked erythema (field size) and dry desquamation; 3 for dry desquamation and dry crusting; 3.5 for dry desquamation, dry crusting, and superficial minimal scabbing; 4 for patchy moist desquamation and moderate scabbing; 4.5 for confluent moist desquamation, ulcer and large deep scabs; and 5 for open wound full-thickness and skin loss^26,36,37^.

### Adipose derived mesenchymal stem cells

Mature female guinea pigs (Hartly Dunkin, n = 5, 450–500 g) were selected and housed within the Laboratory Animal Center of Tarbiat Modares University, Tehran, Iran. The animals were held under regulated temperature (22 °C) in conventional cages, with a 12 h light–dark cycle. Guinea pigs were euthanized by intraperitoneal injection of ketamine 10% and xylazine 2% (Alfasan, Woerden, Holland) and the adipose tissue of the neck site was finely minced, rinsed twice with phosphate-buffered saline, and digested with collagenase 0.1% solution (Gibco; Scotland, Uk). The expansion medium consisted of DMEM (Dulbecco’s Modified Eagle Medium) with 10% FBS, 1% penicillin, and 1% streptomycin. Primary cells were defined as passage 0. During the expansion, the medium was replaced every 2–3 days. In explant method, the adipose tissue was cut into small pieces (1 mm^2^), explanted in a six-well culture plate, and cultured at 37 °C in an incubator with 5% CO_2_ and saturated humidity. The medium was replaced after 48 h. The cells were used in the 3rd passage. Images were taken with an Olympus microscope (ix70, Olympus Co, Tokyo, Japan).

### Differentiation potential of AdMSCs

**Figure 11:**
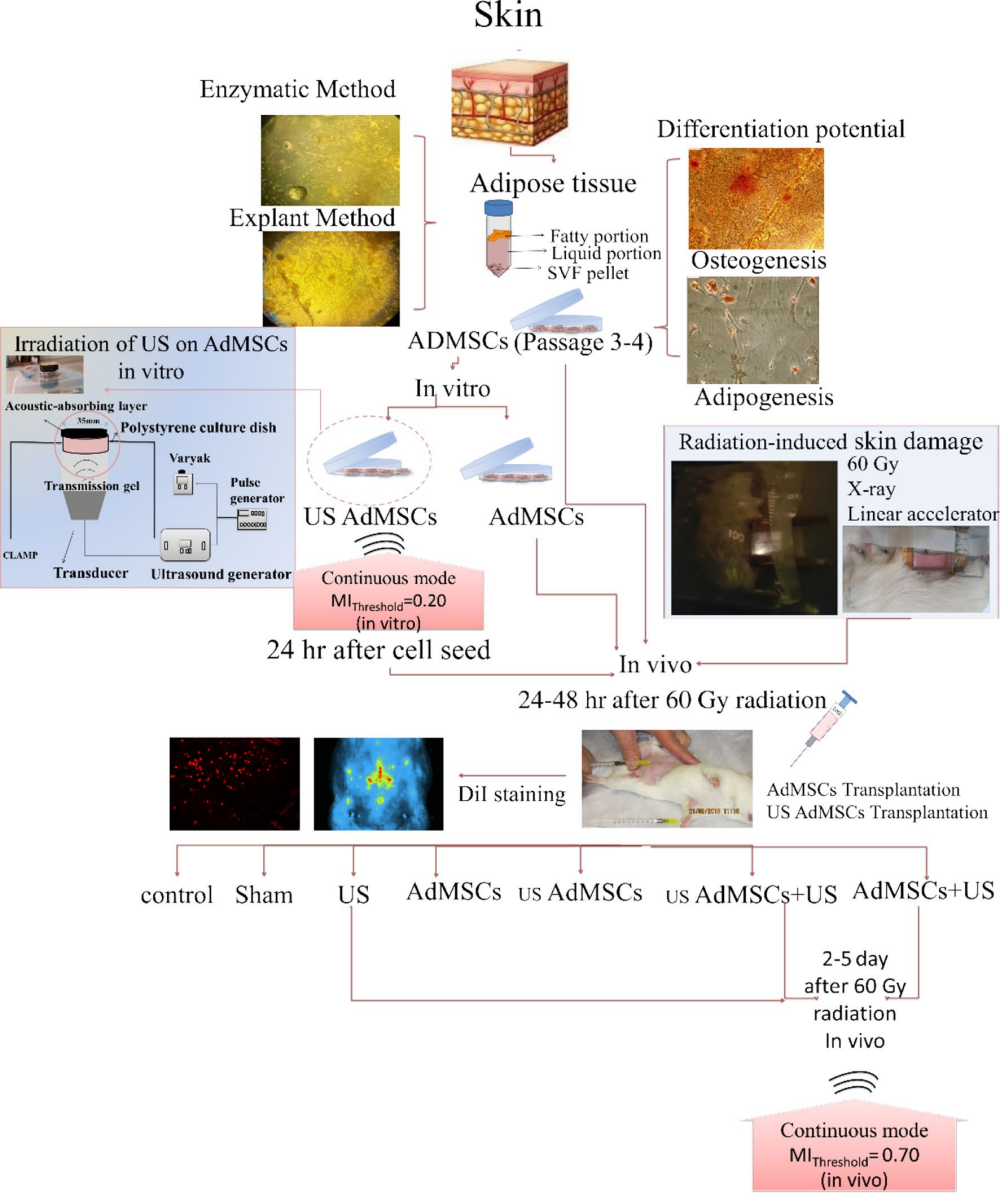
The framework of radiation-induced skin tissue injury treatment by low-intensity ultrasound combined with allogeneic AdMSCs. (US AdMSCs) group: Sonication of AdMSCs with mechanical index parameter (MI = 0.20) in vitro. (AdMSCs + US) group: AdMSCs injection in the first days after 60 Gy radiation and sonication with mechanical index parameter (MI = 0.70) on AdMSCs for three consecutive days in vivo. (US AdMSCs + US) group: Sonication of AdMSCs (MI = 0.20, in vitro) and its injection in the first days after 60 Gy radiation, and also sonication (MI = 0.70, in vivo) on AdMSCs for three consecutive days. Stem cell differentiation and tracking were performed to confirm the nature and presence of stem cells at the radiation- induced skin injury. Results confirmed the presence of AdMSCs on the 7th and 21st days by DiI staining.

The multipotent capacity of AdMSCs to differentiate was performed toward adipogenic and osteogenic cell lineages. AdMSCs were cultured with DMEM, or 10% FBS, until 50% confluence, and then the cells were cultured in the adipogenic and osteogenic medium. The medium was replaced every 2–3 days. Adipogenic differentiation was confirmed after 3 weeks by Oil Red staining to verify the presence of intracellular lipid vacuoles. Osteogenic differentiation, extracellular matrix mineral deposition, and red-stained calcium deposits were confirmed by using an Alizarin Red (Bonyakhte Co, Tehran, Iran) solution (Fig. 11).

### Stem cell tracking

The presence, homing, and migration of AdMSCs in the damaged tissue on the 7th and 21st days after transplantation have been studied through DiI (dialkyclabocyanunes) staining histology of skin tissue and animal imaging (in vivo imaging). The DiI (C7000) staining material was indicated with fluorescent absorption (553 nm) and emission (570 nm) spectra. Images were taken with an Olympus fluorescent microscope (model × 70, Olympus Co, Tokyo, Japan). In vivo imaging was performed using biomolecular imaging (UVItec system, Cambridge, UK). Guinea pigs were anesthetized, and the images were taken seven days after AdMSCs injection.

### Acoustic cavitation threshold

Based on acoustic pressure equations and t the effective radiation area (ERA) of the probe, the interference of the acoustic waves in the environment was modeled. The texture characteristics are including density, speed of sound and attenuation coefficient with 993.37 kg/m^3^, 1528 m/s and 0.025 Np/m MHz in water 37 °C and 1090 kg/m^3^, 1615 m/s, 4.02 Np/m.MHz in skin tissue 35–37 °C, respectively^17^. Then based on the wave interference scheme, the axial transverse profiles of the acoustic pressure in the resulting cylindrical coordinates are solved in programming FORTRAN (Intel Visual Fortran Composer XE 2011) With PC with Core i5-4200 MCPU-2.50 GHz–6 GB RAM. The MATLAB version R2012a (64-bit made by MathWorks, Massachusetts, USA) was used to plot the pressure contours. By solving the pressure equation at each point and extracting the minimum acoustic pressure (*P*_*r*_) specified in the mechanical index (MI) formula to the desired frequency (*f*), the mechanical threshold index at a specified distance is obtained. Non-thermal interactions with the mechanical index parameter are estimated: MI=$\frac{Pr}{\surd f}$^18^. *P* (MPa) represents the negative pressure peak and* f* (MHz) is the frequency. The mechanical index threshold represents the acoustic cavitation threshold that is accord to AIUM defined in water (0.2) and tissue (0.7)^19^. To perform the calculations accurately, the calculations were performed at the appropriate spatial resolution in both axial and radial directions for frequencies of 40 kHz. The minimum wavelength (λ_min_) for the frequency of 40 kHz is 38 mm. Therefore, the minimum wavelength value, the best resolution for this study, is 10^−5^ m.

**Figure 12:**
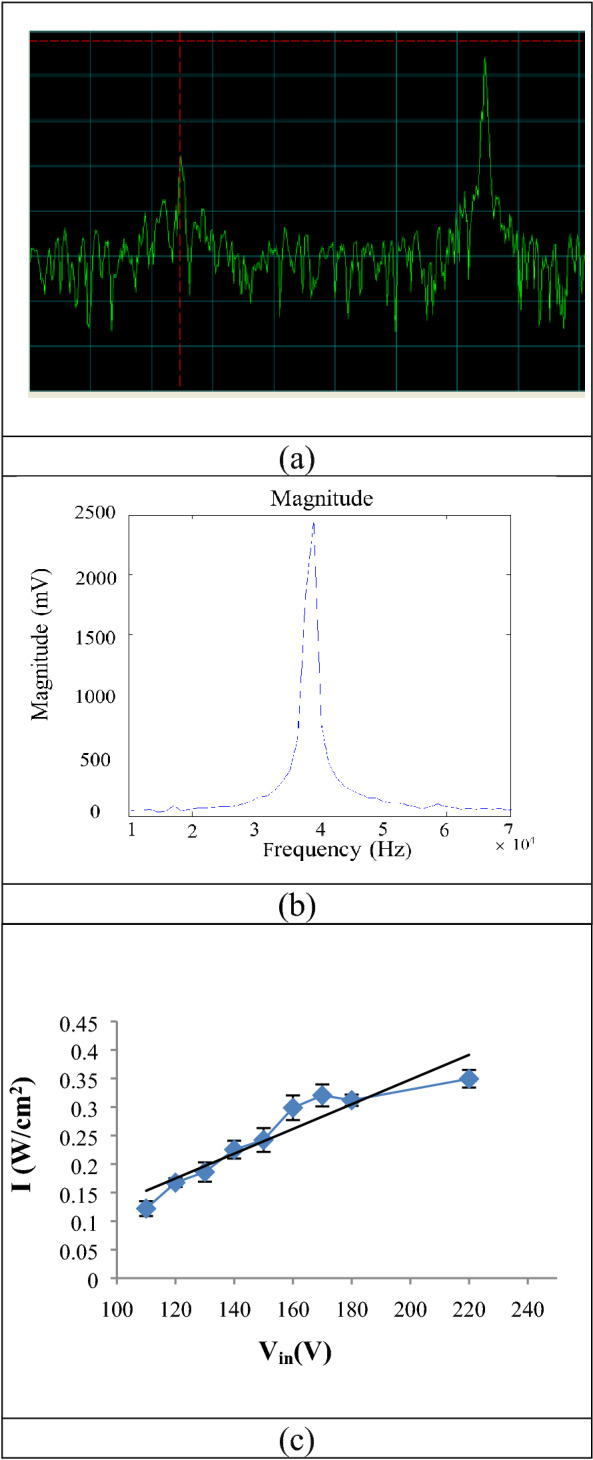
(**a**) The sample of 40 kHz spectrum recorded by a spectrum analyzer, (**b**) the spectrum processed in MATLAB with the specified peak, (**c**) the output intensities of the 40 kHz ultrasound device for different input voltages.

To derive the mechanical index, a low-frequency and intensity ultrasound device was made according to other studies. The output of a 40 kHz ultrasound device (a designed and constructed system in Ultrasound Laboratory, Medical Physics Department, Tarbiat Modares University). The output intensities of the ultrasonic device of 40 kHz for different input voltages were obtained by measuring the output intensities in vitro using a piston hydrophone device (PA124 piston hydrophone, 25 mm diameter, 20 kHz–1 MHz, Precision Acoustics Ltd, Dorchester, UK) (Fig. 12a). The signals recorded for frequency content extraction were analyzed using Fourier Transform Analysis (FFT) in MATLAB software (Fig. 12b). To reduce the error, measurement of the acoustic signal amplitude (mV) was repeated five times in each irradiation condition, the intensity in each group was obtained in W/cm^2^ (Fig. 12c).

### The exposure time of ultrasound

Exposure time was controlled by a micro-thermometer (Multilogger Thermometer CHY/502A, Taiwan, ± 1 °C) during ultrasound stimulation with a continuous mode. To eliminate the ultrasound thermal interaction on the cells and skin tissue, temperature changes (1 °C, lower than the hyperthermia limit) was monitored in the culture medium and the skin tissue. At least three replicates were used for statistical analysis.

### Sonication on cells in vitro

To investigate the effects of ultrasonic radiation on cells before transplantation to improve cell performance, AdMSCs were seeded into an enclosed sterile 3.5 cm tissue culture plate in the 3rd passage. The cells were maintained in DMEM with 10% FBS. The cells were sonicated to a low-intensity ultrasound with a 0.20 mechanical index with the continuous mode in an incubator at 37 °C (in vitro) after 24 h (Fig. 11). After ultrasound treatment, cells were returned to another incubator (37 ºC, 5.3% CO_2_).

### Sonication on cells in vivo

In order to delay the time of injury and prevent its progression in the tissue, AdMSCs (2 × 10^6^ cells) were transplanted 24 h after irradiation of 60 Gy. AdMSCs were injected intradermally using sterile syringes. Low-intensity sonication was used in a continuous mode for three consecutive days in vivo with a 0.70 mechanical index (Fig. 11).

**Figure 13:**
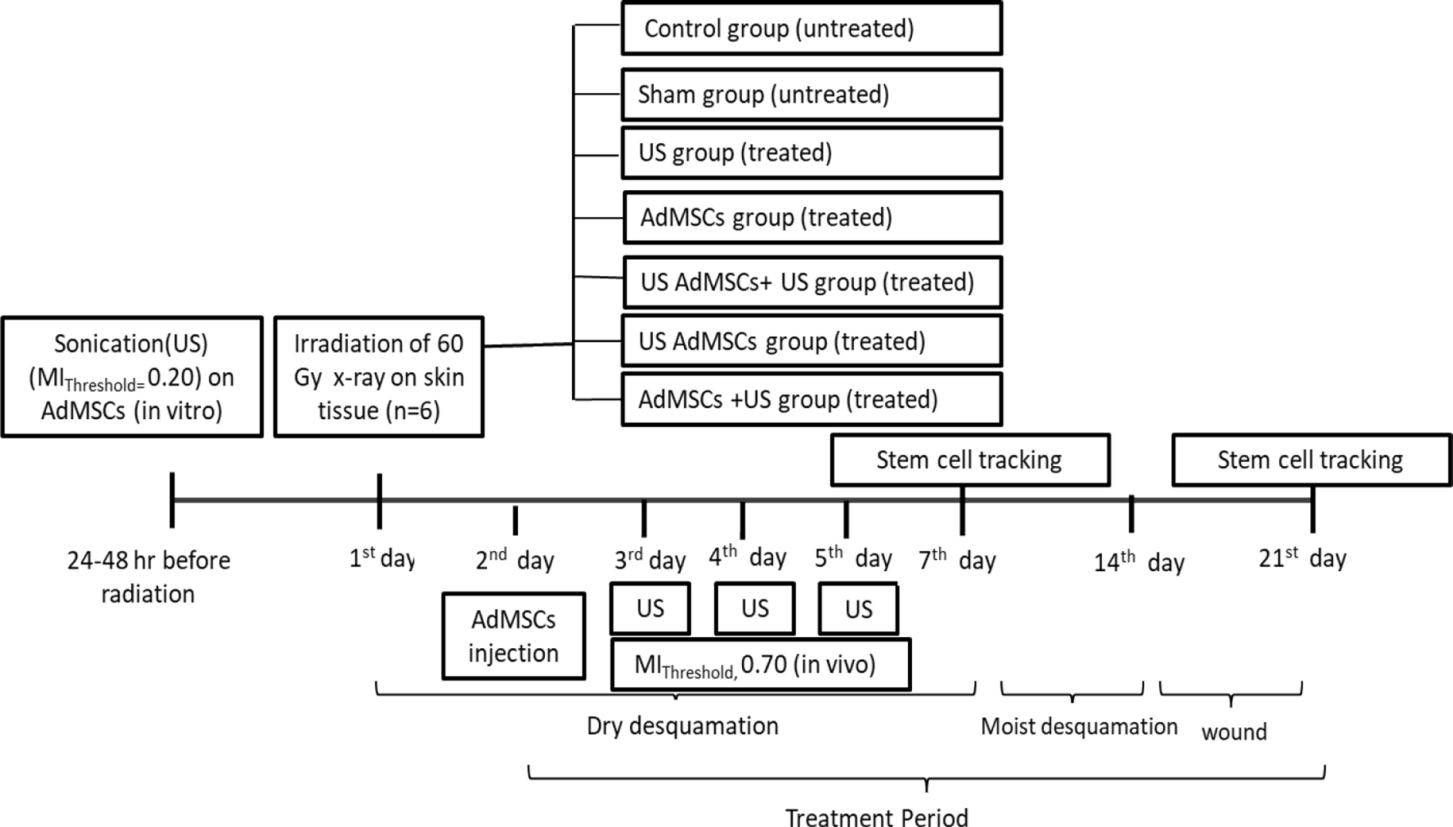
The time period of the combined treatment method to improve acute radiation damage to the skin tissue in the guinea pig model.

First, all animals were exposed to 60 Gy of radiation. Then, the groups were determined as follows: control group: *without any treatment; sham group* treated with normal saline injection; *US group* sonication with a 0.70 mechanical index, 24 h after radiation; *AdMSCs group* treated with AdMSCs; *US AdMSCs group* treated with AdMSCs, under sonication with a 0.20 mechanical index (24 h before transplantation); *AdMSCs* + *US group* treated with AdMSCs transplantation and sonication with a 0.70 mechanical index, 24 h after transplantation; *US AdMSCs* + *US group* treated with AdMSCs transplant under sonication with an optimum mechanical index range, 24 h before and 24 h after transplantation. Six animals were placed in each group (n = 42). Serial photographs were taken as regular measurements of the improvement of acute radiation-induced injuries to skin tissue within 21 days in all groups (Fig. 13). The abdominal skin surface was photographed to determine the macroscopic evidence of acute radiation injury. In the first and second weeks, high-frequency ultrasonography and elastography were evaluated in all groups. In addition, the skin biopsy of all groups was examined for tissue changes in the second and third weeks. At the end of the third week, even tensiometry as an invasive method was also performed (Fig. 13).

### Ultrasound imaging

The size of the skin tissue layers in the ultrasound images in each group was extracted by processing ultrasound images with a frequency of 40 MHz and a resolution of 0.01 mm (Ultrasonix co, Richmond, Canada) via Image J software 1 and 2 weeks after irradiation. The elasticity of the skin layers was determined by ultrasound imaging. High-frequency ultrasonography was taken from three areas, including the left, right, and center of the irradiated area. In each image, the layers’ thickness measurement was performed five times (n = 6 per group).

### Elasticity

The force (0.2 N) was applied by the ultrasonic probe to the skin tissue while simultaneously performing ultrasound and elastography recording. The amount of displacement per force was extracted by the algorithm written in the MATLAB program, and then the elasticity (Young’s modulus) of the skin tissue was estimated in different groups. The ultrasound video was converted into frames in the MATLAB, Then, by selecting the trigger on the skin tissue (epidermis and dermis), the skin layer displacement was calculated for the force applied over time. Variables including the thickness of the skin before applying force L_0Max_ (mm), force F (N), probe area A (mm^2^), and the thickness of the skin after applying force L_Min_ (mm) were extracted. In the Young’s modulus formula $\left(\frac{F\times {\text{L }}_{^\circ (\text{Max})}}{\text{A}\times ({\text{L}}_{(\text{Min})}-{\text{L}}_{^\circ (\text{Max})} )}\right)$, elasticity was expressed in kPa (n = 6 per group).

### Tensiometry

At the end of the treatment, after sacrificing the animals, the abdominal skin tissue of the treatment area was harvested (n = 6 per group). The elasticity of the skin tissue was evaluated by tensiometry as an invasive method to evaluate tissue function and repair. The skin tissue was stretched with a tensiometry machine (Zwick universal testing machine Z2.5, ULM, Germany) until the skin tissue became ripped. Finally, stiffness was evaluated by measuring the linear portion of the stress–strain curve of the abdominal skin specimen. Stiffness (N/mm) equals the value of the slope of this line.

### Morphological analysis of histology

According to similar studies of animals in the treatment, control, and sham groups, the biopsy of skin tissue was collected on the 14th and 21st days of treatment, to evaluate the rate of epithelial healing (n = 6). Hematoxylin and Eosin (H&E) staining was performed to investigate the skin repair and staining of Masson’s trichrome for collagen fibers. Histological images indicated the presence of fibroblast cells, keratinocyte cells, collagen filaments, inflammatory cells, and epithelialization, as well as the formation of the epidermis layer. Wounds were graded according to the semi-quantitative method^38^. This method was used to evaluate following histological processes and structures: epithelization, inflammatory cells, fibroblasts, angiogenesis, and new collagen. Sections were evaluated according to the scale: 0, 1, 2, 3, 4.

### Statistical analysis

The type and severity of skin tissue injury were analyzed using Kumar’s study score on days 1 to 21 after this novel treatment method. The thickness of the epidermis and the dermis layers was analyzed. Functional changes in skin tissue were evaluated with Young’s modulus (kPa) by non-invasive and invasive methods. One-way ANOVA tests were performed to analyze differences between treatment protocols, at a significance level of 0.05 (p < 0.05). The results of the treatment protocol were reported as mean ± SD. A pilot study was conducted for each of the experiments with six animals. In this study, 42 animals were examined during acute skin tissue radiation-induced injury treatment (n = 6). All the data were analyzed by using the SPSS statistical software v 20 (SPSS/PC Inc., Chicago, IL).

### Ethics approval and consent to participate

The animal experiments followed the guidelines of the Laboratory Animal Ethical Commission of the Faculty of Medical Sciences, Tarbiat Modares University. The approval No. IR.TMU.REC.1395.541.

## Data Availability

All relevant data are included within the paper and supporting information files. Please contact the corresponding author for material availability.
